# Comparative evaluation of the anti-Hepatitis B virus activity of *Centella asiatica* and *Camellia sinensis* (green tea)

**DOI:** 10.1186/1471-2334-14-S3-P21

**Published:** 2014-05-27

**Authors:** S Thanigaivel, H Durgadevi, J Balasubramaniam, V Mythily, M Elanchezhiyan

**Affiliations:** 1 Department of Microbiology, Dr. ALM PG IBMS, University of Madras, Chennai, India; 2 Blood Bank, Voluntary Health Services (VHS), Taramani, Chennai, India

## Background

Chronic Hepatitis B virus (HBV) infection is a major health problem and available anti-HBV drugs are known to cause side effects in addition to increased incidence of drug resistance. In the current study anti-HBV properties of *Centella asiatica* and *Camellia sinensis* was evaluated.

## Methods

For the assay, equal volume of HBV virus was mixed with extract of *C. asiatica* or *C. sinensis* and incubated at 37°C for 5 days. The supernatant was assayed for the presence of bound/unbound HBsAg using ELISA. A dose response analysis was done for each extract and cytotoxicity of the each extract was measured by MTT assay.

## Results

Dose response anti HBV revealed that methanolic extract *C. asiatica* indicated that 2.5 mg/mL concentrations was inhibitory to 0.75 pg/mL of HBV. Aqueous and methanolic extract of *C. sinensis* indicated that 0.5 mg/mL and 0.25 concentrations inhibited HBV, respectively. Interestingly, EGCG indicated 1.5 pg/mL HBV was inhibited by 25µg/mL. These concentrations were well tolerated by HepG2 cells and the non toxic concentration was up to 800 µg/mL.

## Conclusion

The study showed that EGCG outperformed other extracts and had anti-HBV activity with minimal concentration (25 µg/mL). This is followed by extracts of *C. sisensis* which exhibited medium anti HBV activity. Poorest anti-HBV activity was noticed with *C. asiatica* i.e.2.5 mg. None of the extracts had cytotoxicity. From this study we could conclude that *C. sinensis* extracts are better in inhibiting HBV.

